# A simple functional marker to predict the need for prolonged mechanical ventilation in patients with Guillain-Barré syndrome

**DOI:** 10.1186/cc10043

**Published:** 2011-02-21

**Authors:** François Fourrier, Laurent Robriquet, Jean-François Hurtevent, Shirley Spagnolo

**Affiliations:** 1 Département Universitaire de Réanimation (Université de Lille 2) et Service de Réanimation Polyvalente, Hôpital Roger Salengro CHRU de Lille, Rue Emile Laine 59037, Lille, France; 2 Service de Neurophysiologie Clinique - Hôpital Roger Salengro CHRU de Lille, Rue Emile Laine 59037, Lille, France

## Abstract

**Introduction:**

Patients suffering from Guillain-Barré syndrome (GBS) may frequently develop an acute respiratory failure and need ventilatory support. Immune therapy using plasma exchange or immunoglobulins has modified the natural course of the disease and by decreasing the length of the plateau phase, may induce a rapid improvement in ventilatory function. However a substantial proportion of patients still require prolonged mechanical ventilation (MV) and tracheotomy. The present study was designed to search for simple functional markers that could predict the need for prolonged MV just after completion of immune therapy.

**Methods:**

We analyzed the data collected in a cohort of patients with GBS admitted to the intensive care unit (ICU) of our university hospital between 1996 and 2009. Demographic, clinical, biological and electrophysiologic data, results of sequential spirometry, and times of endotracheal intubation, tracheotomy, and MV weaning were prospectively collected for all patients. Sequential daily neurological testing used standardized data collection by the same investigators all along the study period. Results were compared by single and multiple regression analysis at admission to ICU and at the end of immune therapy, according to the need and duration of MV (≤ or > 15 days).

**Results:**

Sixty-one patients with severe GBS were studied. Sixty-six percent required MV (median length: 24 days). The lack of foot flexion ability at ICU admission and at the end of immunotherapy was significantly associated with MV length > 15 days (positive predictive value: 82%; odds ratio: 5.4 [1.2 - 23.8] and 82%; 6.4 [1.4 - 28.8], respectively). The association of a sciatic nerve motor conduction block with the lack of foot flexion at the end of immunotherapy was associated with prolonged MV with a 100% positive predictive value.

**Conclusions:**

In patients admitted to ICU with Guillain-Barré syndrome and acute respiratory failure, the lack of foot flexion ability at the end of immune therapy predicts a prolonged duration of MV. Combined with a sciatic motor conduction block, it may be a strong argument to perform an early tracheotomy.

## Introduction

About 30% of patients with Guillain-Barré syndrome (GBS) will require endotracheal intubation and mechanical ventilation (MV) [1,2]. Predictors of the need for intubation and MV at the onset of the disease or at intensive care unit (ICU) admission have been extensively studied. Data from multicenter therapeutic trials comparing plasma exchange (PE) with intravenous immunoglobulins (IVIgs) could document that simple clinical and biological markers could accurately predict these needs. Inability to walk, inability to lift one's head, decreased vital capacity (VC), delay between onset of symptoms and hospital admission of less than 7 days, and high liver enzyme levels were found to have good predictive values [3-7].

Usually, tracheotomy is indicated in these patients when a long duration of MV is expected. However, immunotherapy using PE or IVIgs has changed the natural course of the disease. It decreases the duration of the plateau phase and hastens functional recovery [1-8]. Thus, early weaning from MV may obviate the need for tracheotomy. However, simple clinical predictors of the need for prolonged MV are lacking in these treated patients. Single-center studies have shown that older age and an underlying pulmonary disease were significant risk factors for prolonged MV, but accurate predictors have not been determined [8]. Thus, practically, after admission to the ICU for an acute respiratory failure, these patients receive PE or IVIgs during the first days of their stay. These treatments usually require 5 to 8 days to be completed and the same period of time is needed to evaluate their effect, delaying the decision to eventually perform tracheotomy. Delayed tracheotomy has been shown to increase the risk of severe local and infectious complications, especially ventilator-associated pneumonia [9-11]. On the other hand, when performed too early during the treatment period, tracheotomy may be useless or pose a higher risk.

For 20 years, during which time we participated in the multicenter trials of the French GBS Study Group, patients have been managed in our ICU by the same physicians and therapists in accordance with the same protocols of treatments and repeated functional assessments. To our knowledge, no study has been specifically dedicated to determine, just after completion of immunotherapy, the predictors of a long duration of MV. We designed the present study to search for simple functional markers that could predict the need for prolonged MV.

## Materials and methods

We included in the study all patients who both were referred to the 16-bed ICU of the Roger Salengro University Hospital (Lille, France) between January 1996 and March 2009 and fulfilled standard clinical diagnostic criteria for GBS. The diagnosis was always confirmed by electromyography (EMG), which was systematically performed during the first week after hospital admission. Patients were excluded if they had nonidiopathic GBS. Patients were transferred to our ICU from either an emergency department or a neurological ward. Reasons for admission were usually either a suspected or established acute respiratory failure due to neurological impairment of respiratory muscles.

Patients entering the ICU in acute respiratory distress or hypercapnia were intubated without delay. In the other cases, tracheal intubation was decided when VC measured at the bedside was less than 20% of the predicted value. Patients were ventilated in assist-controlled mode with repeated sequences of assisted spontaneous breathing under physiotherapist supervision. All patients were treated by immunotherapy with either polyvalent high-dose IVIgs or PE (four to six procedures). Each PE procedure allowed the exchange of 1.5 plasma mass, replaced by albumin or plasma substitutes. The IVIg treatment regimen was always 0.4 g/kg of body weight daily for 5 consecutive days. These treatments were completed within 8 days after admission. Additionally, during the ICU stay, patients received symptomatic treatments and nursing care, including systematic anticoagulation to prevent thrombosis, early enteral nutrition, and parenteral vitamins. Tight glucose control was not used.

Throughout the study, the same physicians assessed patients' neurological condition and the same physiologist (JFH) interpreted EMG data. Respiratory function was assessed by repeated spirometry performed at the bedside. Spirometric measurements were done by the same well-trained respiratory physiotherapists and included minute ventilation, respiratory frequency, VC, and maximal minute ventilation [12].

Weaning from MV was decided on the basis of the results of clinical assessment and sequential spirometric measurements. Weaning procedures followed the criteria and recommendations of the 8th and 21st French Consensus Conferences in ICU and Emergency Medicine [13,14]. Weaning and extubation were usually decided when VC was greater than 20% of the predicted value and maximal minute ventilation was twice the minute ventilation, after a 3- to 6-hour trial of T-tube ventilation. The physicians in charge made the decision to perform tracheotomy on the basis of the patient's status, usually when no functional and respiratory improvement was observed within 10 days after completion of immunotherapy.

The following characteristics were prospectively collected for all patients: age, sex, severity of illness at admission as assessed by the Simplified Acute Physiology Score II (SAPS II), delays from GBS onset to hospital admission and ICU admission, and ICU length of stay. Neurological testing used the standardized data collection elaborated by the French Cooperative Group on Plasma Exchange in GBS [4]. The following data were noted: (a) weakness of the limbs, (b) deep tendon reflex abolition in the upper and lower limbs, (c) cranial nerve impairment, (d) weakness of trunk and respiratory muscles (VC level and ability to lift and hold one's head above the bed), (e) presence of paresthesia or impaired vibratory sensitivity in the limbs, and (f) cardiovascular autonomic dysfunction defined as an increase or decrease (40 mm Hg) in systolic blood pressure, spontaneous or induced bradycardia (heart rate decrease of greater than 20 beats per minute), or spontaneous tachycardia (increase to greater than 120 beats per minute without fever). Cerebrospinal fluid analysis and the following biochemical tests were performed in all patients at ICU admission: natremia, glycemia, creatine phosphokinase, liver enzyme, platelet count, prothrombin time, and C-reactive protein levels. Standard viral serology, including cytomegalovirus and *Campylobacter jejuni*, was realized. Clinical and laboratory data were collected daily until the plateau phase was recognized and thereafter when significant neurological changes were observed. To search for significant neurological improvement or worsening, changes in motor scores were analyzed for all patients between admission and completion of immune therapy. Neurological improvement or worsening was defined as the reappearance or the disappearance of a spontaneous perceptible movement in one of the items of the standardized neurological functional testing. The definite functional outcome was assessed by walking ability (independent ambulation, requiring assistance to walk, or bed-bound) at a follow-up medical consultation 4 to 6 months after discharge from the ICU. Data from electrophysiological testing were analyzed to classify GBS as acute motor axonal neuropathy, acute motor and sensory axonal neuropathy, acute inflammatory demyelinating polyradiculoneuropathy (AIDP), or AIDP with axonal involvement. Motor conduction block for sciatic nerves was defined as an at least 50% decrease in amplitude of the compound muscle action potential (CMAP) [15].

For the purpose of this study, we compared the patients according to the need of MV to verify whether our population was representative of most GBS cases and exhibited previously known prognostic markers. Then the group of ventilated patients (MV^+ ^group) was specifically studied, and the data collected at ICU admission, at the end of immunotherapy, and 8 days after completion of immunotherapy were extracted for each patient. The following time periods were calculated: time from ICU admission to tracheal intubation, time from the end of specific treatment to MV weaning, duration of MV, and timing of tracheotomy. MV weaning was defined as definite extubation or, in tracheotomized patients, when spontaneous ventilation was obtained 24 hours daily. Finally, two subgroups were defined according to the duration of MV (≤ or >15 days) and all of their clinical and biological data were compared to seek for significant predictors of prolonged MV. Prospective collection of patients' data was approved by the ethics committee of our university hospital in 1996. Since this was a fully retrospective study with all patients' data rendered anonymous and according to French law, informed consent was not requested.

### Statistical methods

Descriptive analysis (frequencies for categorical data, mean ± standard deviation, median with ranges for numerical variables) was performed in the total population and compared according to the need of MV and to its duration (≤ or >15 days). Categorical variables were compared by the chi-square test or the Fisher test when appropriate. Continuous variables were compared by the Mann-Witney Whitney test. Differences between groups tested by univariate analysis were considered to be significant for variables yielding a *P *value of less than 0.05. Variables attaining a 0.05 α value were included in a multiple logistic regression analysis model with stepwise selection. All statistical analyses were performed with the SAS Software, version 8.2 (SAS Institute Inc., Cary, NC, USA).

## Results

### Characteristics of the studied population

Between January 1996 and March 2009, 61 patients were admitted to our ICU for idiopathic GBS. The main demographic data are shown in Table 1. Demyelinating forms of GBS were predominant (75%). The mean age was 51.9 ± 20.5 years, and males were slightly predominant (54%). The mean SAPS II was 23 ± 16 points. The median ICU length of stay was 19 days (range of 1 to 549). Overall, four patients died in the ICU from sudden cardiac arrest due to severe autonomic dysfunction (*n *= 3) or from septic shock (*n *= 1). The mortality rates were 6.5% in the whole population and 10% in the ventilated group. Fifty-seven patients were discharged from the ICU, and 40 patients could be assessed during a follow-up consultation after a median delay of 5 months. At that time, 5% of the patients were bed-bound, 75% could walk independently, and 20% with assistance.

**Table 1:** Characteristics of the studied population (*n *= 61)

Characteristics	Results
Age, years	51.9 ± 20.5
Sex, males/females	33/28
SAPS II	23.1 ± 16.8
DSH, days	5.8 ± 6.3 (median delay: 3)
Delay between onset of symptoms and ICU admission, days	9.8 ± 10.4 (median delay: 6)
Mechanical ventilation, number (percentage)	40 (65)
Tracheotomy, number (percentage)	28/40 ventilated patients (70)
ICU length of stay, days	40 ± 74 (median length: 19)
EMG type of neuropathy, number (percentage)	
AMAN	6 (10)
AMSAN	9 (15)
AIDP	33 (54)
AIDP and axonal involvement	13 (21)
Positive serology, number (percentage)	
Cytomegalovirus	9 (14)
*Campylobacter jejuni*	14 (23)
Neurological presentation at admission to the ICU, number (percentage)	
Severe dysautonomy	19 (31)
Bilateral facial nerve palsy	25 (41)
Inability to lift one's head above the bed	22 (36)
Bilateral lack of forearm flexion	23 (38)
Bilateral lack of elbow elevation above bed	24 (56)
Bilateral lack of foot flexion	29 (48)
Bilateral lack of foot extension	28 (46)

Data are presented as mean ± standard deviation unless otherwise stated. AIDP, acute inflammatory demyelinating polyradiculoneuropathy; AMAN, Acute Motor Axonal Neuropathy; AMSAN, Acute Motor and Sensory Axonal Neuropathy; DSH, delay between onset of symptoms and hospital admission; EMG, electromyography; ICU, intensive care unit; SAPS II, Simplified Acute Physiology Score II.

### Group comparisons

**Figure 1:**
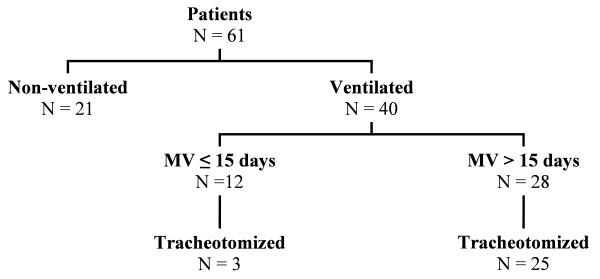
**Flow chart of the study**. MV, mechanical ventilation.

The study flow chart is shown in Figure 1. Forty patients (66%) required MV with a median duration of 24 days (range of 6 to 540). The median delay between ICU admission and intubation was 2 days. Tracheotomy was performed in 28 patients (median delay between tracheal intubation and tracheotomy: 14 days). In three patients, tracheotomy was performed within days after admission since they did not tolerate the intubation tube (one patient) or were considered at high risk of extubation because of severe hypoxemia and autonomic dysfunction (two patients). These three patients survived and were weaned before day 15. Among the 19 tracheotomized patients who were assessed at a follow-up visit, complete weaning and tracheal tube removal had been obtained in 14 cases.

The comparison between ventilated (MV^+^) and nonventilated (MV^-^) patients was done to verify the presence of the usual markers of severity in the studied population. The main results are shown in Table 2. MV^+ ^patients had a higher SAPS II (*P *= 0.0052) and a longer ICU length of stay (*P *< 0.001). Glycemia at admission was significantly higher in the MV^+ ^group (8.07 versus 6.51 mmol/L; *P *< 0.01). The delay between immunotherapy and neurological improvement was longer (*P *< 0.001). As expected, the frequency of severe autonomic dysfunction and acquired infections was dramatically higher in the MV^+ ^group. When studied with a multiple logistic regression model, the risk of MV was significantly increased when the patient exhibited at least one sign of cardiovascular autonomic dysfunction and was unable to lift his or her head above the bed (odds ratio 10.66, 95% confidence interval [CI] 2.4 to 49; *P *< 0.05 and 9.86, 95% CI 1.7 to 56; *P *< 0.05, respectively). Concerning final functional outcome, walking autonomy was observed without significant difference between patients requiring MV or not. These results were consistent with a 'standard' population of patients with GBS.

**Table 2:** Comparison of patients according to mechanical ventilation requirement

Characteristics	Ventilated (MV^+^) patients *n *= 40	Nonventilated (MV^-^) patients *n *= 21	*P *value
Sex, males/females	23/17	10/11	NS
Age, years	56 ± 20	46 ± 19	NS
SAPS II	28.2 ± 18.6	14.1 ± 6.8	0.0052
DSH, days	5.9 ± 6.9	5.8 ± 5.1	NS
Autonomic dysfunction, percentage	60	14	0.0006
Intensive care unit length of stay, days	40.4	13.0	0.001
EMG subtype, percentage of demyelinating type	77	71	NS
Infectious complications, percentage	85	5	0.0001
Median delay between immunotherapy and neurological improvement, days	27	4	0.001
Independent ambulation at follow-up, percentage	50	58	NS
Deaths, number (percentage)	4 (10)	0 (0)	-

Data are presented as mean ± standard deviation (median) unless otherwise stated. DSH, delay between onset of symptoms and hospital admission; EMG, electromyography; MV, mechanical ventilation; NS, not significant; SAPS II, Simplified Acute Physiology Score II.

To determine the predictors of prolonged MV, patients in the ventilated group were compared according to the duration of MV (≤ or >15 days). The main results are shown in Table 3. There was no demographic difference concerning gender, age, SAPS II, frequency of cytomegalovirus and *C. jejuni *infections, and frequency of demyelinating GBS subtype. Differences in neurological testing by standardized data collection were searched at two time points: admission to the ICU (day 1) and end of immunotherapy (day 8 to 10). Owing to reduced sample size, multiple logistic regression analysis was not possible. At the ICU admission time point, only three variables were significantly different in patients who subsequently needed more than 15 days of MV: the median level of VC was significantly lower (46% versus 63% of the predicted value; *P *< 0.01), plasma sodium concentration was significantly lower (*P *< 0.05), and foot flexion ability was more frequently lacking (35% versus 66%; *P *< 0.004). No difference could be observed in any of the other neurological clinical parameters. At the end of immunotherapy, no difference was observed in any of the measured clinical parameters, except for foot flexion ability and presence of a sciatic motor conduction block. Worsening of the neurological motor situation was observed in six patients in the group ventilated more than 15 days. In five cases, it concerned the foot flexion ability, which disappeared between days 1 and 10. Worsening was observed in only one patient in the group ventilated less than 15 days. The operating characteristics of the differences in frequency of foot flexion ability and motor conduction block were calculated at the admission time point and at the end of immune therapy (Table 4). At admission to the ICU and at the end of immune therapy, lack of foot flexion ability predicted a duration of MV of more than 15 days with a positive predictive value of 82% (odds ratio 5.4, 1.2 to 23.6; *P *< 0.02 and 6.4, 1.4 to 28.8; *P *< 0.01, respectively). A sciatic nerve conduction block was associated with the lack of foot flexion ability in 71% of cases (*P *< 0.02). Conversely, the lack of conduction block was associated with the persistence of foot flexion in 64%. The presence of a conduction block predicted the need of prolonged MV with a positive predictive value of 85% (odds ratio 6.8, 1.4 to 33.5; *P *< 0.05). The combination of a sciatic nerve conduction block with the lack of foot flexion at the end of immunotherapy was associated with a prolonged MV with a specificity of 100%.

**Table 3:** Comparison according to the duration of mechanical ventilation

	MV ≤15 days *n *= 12	MV >15 days *n *= 28	*P *value
Sex, males/females	7/12	16/28	NS
Age, years	50 ± 20	56 ± 21	NS
SAPS II	35 ± 25	25 ± 15	NS
DSH, days	5.0 ± 6.3	6.2 ± 7.2	NS
Delay to improvement, days	12 ± 11	28 ± 30	0.03
ICU length of stay, days	17 ± 10	75 ± 99	0.002
Sodium at admission, mmol/L	136 ± 3.9	133 ± 3.5	0.04
Dysautonomy, number	8/12	16/28	NS
EMG subtype, percentage of demyelinating subtype	63	82	NS
Acquired infection, percentage of patients	75	96	NS
Lack of foot flexion, number (percentage)			
At admission	4 (34)	18 (65)	0.01
At the end of immunotherapy	5 (42)	23 (82)	0.001
Independent ambulation at follow-up, percentage	53	49	NS
Deaths, number	2	2	-

Data are presented as mean ± standard deviation (median) unless otherwise stated. Delay to improvement refers to delay between end of immunotherapy and first neurological improvement. DSH, delay between onset of symptoms and hospital admission; EMG, electromyography; ICU, intensive care unit; MV, mechanical ventilation; NS, not significant; SAPS II, Simplified Acute Physiology Score II.

**Table 4:** Operating characteristics for prediction of prolonged mechanical ventilation (*n *= 40)

Predictive factor	Sensitivity	Specificity	PPV	NPV	Youden index	Odds ratio (95% CI)	*P *value
Lack of foot flexion ability at admission	0.73	0.66	0.82	0.53	0.40	5.4 (1.2-23.6)	<0.02
Lack of foot flexion ability at end of immune therapy	0.82	0.58	0.81	0.58	0.40	6.4 (1.4-28.8)	<0.01
Presence of a motor conduction block	0.72	0.71	0.85	0.53	0.45	6.8 (1.4-33.5)	<0.05
Presence of a motor conduction block and lack of foot flexion ability at end of immune therapy	0.56	1	1	0.39	0.56		<0.001

CI, confidence interval; NPV, negative predictive value; PPV, positive predictive value.

## Discussion

Our study shows that, in patients with GBS, a long duration of MV may be predicted by means of a simple functional marker. In our patients, the lack of foot flexion ability at the end of immunotherapy was significantly associated with an MV length of greater than 15 days. The sensitivity of this simple marker was enhanced by the results of EMG assessment; conversely, the association of a sciatic nerve conduction block with the lack of foot flexion seemed highly specific of a prolonged duration of MV. We think this result may help clinicians to decide whether or not tracheotomy should be performed after completion of immunotherapy.

The patients whom we have studied were representative of severe GBS. Demographic characteristics concerning age, sex ratio, and neurological presentation were in accordance with most published series [5-7,10,11]. Our study confirms the previously known early predictive factors of MV, including inability to lift one's head and a decrease in VC [4,6,8,16,17]. As expected, severe autonomic dysfunction and inability to lift one's head above the bed at ICU admission was were associated with an increased risk of MV requirement. In our study, comparison of SAPS II between ventilated and nonventilated patients showed a significantly higher physiological severity in patients needing MV, but there was no score difference according to the duration of MV.

Since the length of MV in these patients is hardly predictable, finding predictive factors of prolonged mechanical support would help their management. In a multicenter randomized study including medical ICU patients, Rumbak and colleagues [9] concluded that early tracheotomy in those projected to need MV for more than 14 days was associated with a reduction of acquired pneumonias, sedation, length of MV and stay in the ICU, and finally a reduction of hospital mortality. In the standard ICU setting, tracheotomized mechanically ventilated patients needing prolonged MV require less sedation, appear more comfortable, and achieve more autonomy earlier [18,19]. Thus, knowledge of predictive factors of prolonged mechanical support could help clinicians to select GBS patients for early tracheotomy.

To our knowledge, the study by Lawn and Widjicks [8] is the only one that could determine criteria of prolonged MV in GBS patients. These authors described a score combining VC and maximal expiratory and inspiratory pressures, measured at admission and 12 days later. In their study, an improvement in this pulmonary functional score predicted an MV duration of less than 3 weeks. In our study, we did not sequentially measure VC and maximal pressures and consequently cannot compare our results with those of Lawn and Widjicks. We found that the lack of foot flexion ability observed at ICU admission and at the end of immunotherapy was significantly more frequent in patients who needed a prolonged duration of MV. Nearly 20% of these patients lost their foot flexion ability between days 1 and 10, giving confirmation that neurological status should be reassessed just after completion of immune therapy. Additionally, at that time, the combined observation of a sciatic nerve motor conduction block with the lack of foot flexion ability was significantly associated with a prolonged MV with 100% specificity. This should strongly argue in favor of performing tracheotomy when these two criteria are present. Durand and colleagues [7] found, in keeping with our findings, a significant association between respiratory failure and neurological impairment of inferior limbs. The authors showed that peripheral neurophysiological testing was helpful for assessing the risk of respiratory failure. The highest risk of respiratory failure was observed in patients who exhibited a decrease in the CMAP between the proximal and the distal common peroneal nerve. The cutoff point found by Durand and colleagues was a 20% decrease in CMAP. In our study, the diagnosis of motor conduction block of the sciatic nerve was considered for a 50% decrease. This higher severity could explain why it may represent a good predictor of prolonged MV in our study. However, there is no obvious link between the presence of a peripheral conduction block and prolonged diaphragmatic weakness. Severe demyelination is characterized by a pronounced decrease in motor nerve conduction velocities, increase in distal motor latencies, or presence of a conduction block [15]. In patients with GBS, the prolonged impairment of foot flexion is probably evidence of a severe neurological injury with either a major demyelination process or a secondary axonal loss. This feature will reflect a more diffuse neurological injury or an impaired process of nerve recovery affecting phrenic nerves as well.

Our study has clear limitations, which are linked mainly to its retrospective and monocentric design. Only patients with the more severe form of GBS were admitted to our ICU, and our results cannot be extended to less severe types of GBS. Despite these limitations, we think that our population accurately reflects most patients with severe GBS. The long recruitment period between 1996 and 2009 is unlikely to have biased our results, because during this time, procedures of immunotherapy for GBS did not change, and decisions of tracheal intubation, tracheotomy, and MV weaning were made by using the same criteria by the same physicians according to the results of standardized neurological monitoring. We found the same risk factors of MV requirement as reported in published data, giving confirmation of the similarity of our patients with populations of GBS included in preceding larger studies. However, owing to the low number of patients, the operating characteristics of the functional markers predicting the need for prolonged MV should be considered cautiously.

## Conclusions

We conclude from our results that, at the end of immunotherapy, the lack of foot flexion ability associated with a sciatic nerve EMG conduction block may predict a long duration of MV and represent a strong argument to perform tracheotomy. These results should be confirmed by a prospective study or a comparable multicenter retrospective study.

## Key messages

• In patients with Guillain-Barré syndrome and acute neuromuscular respiratory failure, immunoglobulin or plasma exchange therapy decreases the length of the plateau phase and hastens functional recovery. Prediction of a long duration of mechanical ventilation may facilitate patients' management and the decision to perform tracheotomy.

• In our study, the lack of foot flexion ability at intensive care unit admission and at the end of immunotherapy was associated with a prolonged duration of mechanical ventilation.

• The combination of a sciatic nerve conduction block with the lack of foot flexion at the end of immunotherapy was associated with prolonged mechanical ventilation with a positive predictive value of 100%.

## Abbreviations

AIDP: acute inflammatory demyelinating polyradiculoneuropathy; CI: confidence interval; CMAP: compound muscle action potential; EMG: electromyography; GBS: Guillain-Barré syndrome; ICU: intensive care unit; IVIg: intravenous immunoglobulin; MV: mechanical ventilation; PE: plasma exchange; SAPS II: Simplified Acute Physiology Score II; VC: vital capacity.

## Competing interests

The authors declare that they have no competing interests.

## Authors' contributions

SS studied all patients' files, collected data, and partially wrote the paper. LR participated in the design and data interpretation. J-FH reviewed electromyographic data and interpretation. FF designed the study protocol, revised all patients' files and rough data, interpreted the results, and wrote the paper. All authors read and approved the final manuscript.
